# The Obstetrical Remembrancer; or Denman’s Aphorisms on Natural and Difficult Parturition; the Application and Use of Instruments, &c.

**Published:** 1848-06

**Authors:** 


Art. VIII.—*The Obstetrical Remembrancer; or Denman's Aphorisms on Natural and Difficult Parturition; the Application and Use of Instruments,* &c. Augmented by Michael Ryan, M.D. First American from ninth London edition, with additions by Thomas F. Cock, M.D., Visiting Physician to the New York Lying-in Asylum. pp. 258.

*Memoranda on Anatomy, Surgery, and Physiology.* By Mark Noble Bower, Surgeon. Corrected and enlarged by an American Physician. pp. 325.

*Ophthalmic Memoranda respecting those Diseases of the Eye which are more frequently met with in practice.* By John Foote, Fellow of the Royal College of Surgeons. pp. 131.

These books are all from the press of the Messrs. Woods, New York, and are part of a series which that house is publishing. They are intended as medical class-books, and are of a size and shape convenient for the pocket. They will unquestionably prove useful to students, and now and then to practitioners. C.

